# Clinical Supervision and Support: Exploring Pre-registration Nursing Students’ Clinical Practice in Malawi

**DOI:** 10.29024/aogh.16

**Published:** 2018-04-30

**Authors:** N. C. Kaphagawani, U. Useh

**Affiliations:** 1 Malawi College of Health Sciences, Zomba, MW; 2 Faculty of Agriculture Science and Technology, North West University, Mafikeng Campus, Mmabatho 2735, North West Province, ZA

## Abstract

**Background:**

Supervised clinical practice plays a significant role in the nursing profession, as it has an influence on the students’ clinical learning.

**Objectives:**

The aim of this study was to explore how the pre-registration nursing students find their experience on clinical supervision in the clinical placements.

**Methods:**

The study used both quantitative and qualitative approach to collect data through focus groups (n = 144) and self-administered questionnaires (n = 590) from nursing students of various programmes in selected colleges in Malawi.

**Results:**

About 75% (n = 443) of the participants indicated that they received supervision from both clinical staff and Nurse Educator. However, qualitative results indicated that students received inadequate clinical supervision. Themes that emerged from the discussion included lack of human resources, learning support, availability of instructors yet not supporting learning, job insecurity and lack of remuneration as reasons for lack of supervision, role models and student guidance despite pressure and self-directed.

**Conclusion:**

There is a need for clear policies regarding clinical supervision as well as a structured and well monitored process.

## Introduction and Background

Supervised clinical practice plays a significant role in the nursing profession, as it has an influence on the students’ clinical learning of knowledge and skills. Accordingly, clinical supervision promotes the well-being of the students, positive attitudes towards professional development and assists in the need for lifelong learning [9]. It is also known that in order for nursing students to be adequately prepared for practice, they need to be guided and supervised [1,23] Previous studies have revealed that students lack clinical teaching while in clinical practice resulting in learning without guidance [19,20]. This may have a negative effect on their learning in the clinical practice. For example, students may not only be incompetent in their nursing skills but may also have a negative attitude towards the profession. In cases such as these where there is a shortage of nursing educators and clinical staff, nursing students may receive inadequate clinical teaching and supervision, thereby hindering their clinical learning.

## Literature review

Clinical supervision is when a professional expert provides support, guidance and feedback to nursing students or novice nurses to develop skills of nursing practice. Several studies maintain that clinical supervision is an important element in facilitating learning in the clinical setting [22,10,5].

Some authors assert that clinical supervision enhances integration of theory and practice, personal and professional growth, provides support and reduces errors thus ensuring patients’ safety [11,16]. The nurse educator’s role is to enhance learning through provision of opportunities for learning, supporting, guiding and conducting timely and fair evaluations. However, this role is not fulfilled as nurse educators take a role more of evaluation than of supervision, which is mainly done by nursing staff who lack teaching experience and may not know the needs of the students [26]. In addition, heavy workloads and attitudes of staff may compromise supervision [4]. Clinical performance increases if students are given necessary support in the clinical environment [6].

More than one study points out the importance of supervisory relationships in facilitating students’ learning in the clinical environment [28,22,25]. In eight European countries [28] found that students were satisfied with supervisory discussions and mentor ship which provided individualized supervision. This was also emphasized by [22]. However, variations on supervisory models from country to country were evident in the study, as some of the European countries were still using group supervision [28]. Individualized supervision facilitates learning on the premise that a one on one relationship with the mentor or preceptor allows students to talk about their learning experiences and feelings in the practice, thus leading to self-confidence, promotion of role socialization, professional development and independence, thereby attaining clinical competency [22,28].

Contrary to one-on-one supervision, studies have revealed that some students prefer group supervision and cluster facilitation as it promotes their personal and professional growth [11,27]. This suggests that other factors may play a role in facilitating the supervision of students. One study [19] revealed that students were not sufficiently supervised when performing nursing activities. This implies that the students are not provided with specific learning situations, guidance, clear directions, guidelines and feedback on their practical experience, which may have a negative impact on learning (Benner, 2004). The nurse educators play a significant role in facilitating clinical learning of students in the clinical environment. The nurse educators have to work with mentors, providing them with support, direction and information about the students’ theoretical learning and their needs.

There have been inadequate adaptations of the newly graduated nurses, regardless of whether they are educated using a Registered Nurse (RN) model or a Nurse Midwife Technician (NMT) (Enrolled nurse) model in Malawi. Anecdotally, qualified nurses show high levels of poor patient care, limited clinical reasoning and rational judgement to handle clinical complicated cases [21]. Additionally, such lack of critical thinking creates an imbalance in which the nurses are expected to think to meet new dimensions of the changing environment [13,20]. The Nurses and Midwives Council of Malawi report indicated that the challenges are due to the increased numbers of nursing students admitted by the institutions responsible for educating student nurses. In addition, the report indicated that levels of congestion in clinical learning environments coupled with inadequate Nurse Educators (NEs) and other clinical nursing staff. In view of these seemingly related challenges, students may receive inadequate support and supervision, and this negatively affects their clinical learning. Several studies [7,20,18] postulate the importance of clinical supervision for nursing students to acquire knowledge and skills in clinical practice for them to become competent practitioners after graduation.

Despite numerous literature on the clinical supervision of students, it is scanty in Malawi. Therefore, the study sought to explore and describe nursing students’ experiences of clinical supervision in four referral hospitals in Malawi. Hence, it will contribute to the level of knowledge on clinical supervision of nursing students in order to improve students’ clinical learning thereby enacting an effective performance after the students qualify.

## Purpose of the study

The aim of this study was to explore the students’ experiences of the clinical environment on clinical supervision in in Malawi.

### Objectives

– To determine the quality of clinical supervision nursing students received in the clinical settings
– To explore nursing students from different colleges, programmes and year of study placed in the four referral hospitals how their experience was on clinical supervision
– To determine nursing students’ satisfaction with clinical learning

## Methods

A descriptive and exploratory design was used to collect both quantitative data, through a self-administered questionnaire, and qualitative data, through focus group discussions among the nursing students in eight selected colleges in Malawi. The study was based on Kolb’s Experiential Learning theory which is part of experiential education [14]. Recognizing that nursing is a practice-based profession; Experiential Learning Theory (ELT) emphasizes learning by doing and reflection. For this reason, ELT can assist students effectively to learn clinical nursing skills as well as attain educational objectives and increased understanding of the course content, having a positive impact on their learning. The use of these theories as a framework in this study will enhance nursing students’ clinical learning, while giving them the support, encouragement, information, and skills to be effective so that they acquire knowledge, skills, and attitudes to enable them to become competent after qualifying and able to provide quality care.

### Setting

The study was conducted in Malawi, which is situated in Southern Africa. Malawi is divided into four administrative regions, namely: the Northern, Central, Southern and Eastern regions. There are 14 nursing colleges in Malawi and a purposive sampling was used to select eight nursing colleges located in all the regions of Malawi. Purposive sampling was used because the colleges are mainly Christian Health Association of Malawi (CHAM is an umbrella of all Christian health training and service provider institutions) and public institution which offer Bachelor of Science in Nursing, Diploma for Registered Nurses and Diploma in Nursing and Midwifery Technician.

### Study population and sampling

Simple random sampling methods were used to obtain a sample of the participants from the 8 colleges who had finished their clinical experience in referral hospitals. This method allowed all nursing students in the sampled nursing colleges to have an equal chance of being selected into the sample, therefore making the sample representative and the results generalizable to the entire population of nursing students and colleges (Parahoo, 2014). All students in first, second and third years were used as a sampling frame. A Table of random numbers obtained from the appendices of Burns and Grove (2005) was used to select participants. Class registers were used where each student was assigned a number. The researchers used a random number table to select who would be in the study. The individuals whose numbers corresponded to the random numbers were drawn and included in the sample (Burns and Grove, 2005). However, participation in the study was voluntary. Seven hundred pre-registered nursing students in first, second and third years pursuing a qualification of a Bachelor’s degree, a diploma in registered nursing and Nurse Midwife Technicians (NMT) were invited to participate in the study. The researchers chose to include first year students due to their earlier participation in clinical practical activities. A total of 590 students answered the questionnaire representing a response rate of 84%.

The questionnaire was administered to participants after they had completed clinical practice which ranged from two (2) weeks to twelve (12) weeks in different clinical specialities in the referral hospitals. Criteria for participation was that students must have completed clinical placement not more than two weeks before data collection, so that the memory of the experience was still fresh in their minds, therefore the Hawthorn effect was reduced and the validity of the study was strengthened [24]. Students studying post basic courses were excluded as their previous experience may have had an influence on the findings.

The formula below illustrates how students were selected at each chosen college into our sample.

Survey\ student\ participants = \frac{{Total\ Enrolled\ Students}}{{Total\ Number\ of\ Student\ Nurse\ in\ Malawi}}\,\, \times Sample\ size.

Illustratively, College 2 which had a student population of 67 students of which 9 student participants were selected, the following computation was applied to derive to total student participants at the college.

Nonetheless, the sample size was reached to 590 as 10% non-despondence rate was taken into consideration. Additionally, after analysing the pilot study results the sample was small for the method used in analysis. To have an adequate sample for the study, the computed sample size was doubled.

Focus group discussions were used to collect qualitative data. This study comprised of sixteen focus groups. There were two focus groups from each college, with nine students per group three from each year of study which were selected through purposive sampling and a total of 144 students participated in this study. Alternatively, literature recommends the use of four to six focus groups to achieve data saturation (Bryman, 2012, Jayasekara, 2012). The sample size was determined by the saturation of information achieved in order to gain a deeper understanding on clinical supervision. Saturation of data was achieved when the students no longer provided any new information during focus group discussions.

### The questionnaire

The structured questionnaire was developed in English by the researcher and items included were informed from the literature [12,3]. The items on the questionnaire included socio demographic information. In addition, Likert scale items were included which measured students’ perceptions on aspects of clinical supervision. The questionnaire consisted of agreement Likert scale with 17 items grouped into 5 which measured clinical supervision. The items had four Likert scaled responses namely; strongly agree = 4 agree = 3, disagree = 2) and strongly disagree = 4. Participants were asked to respond to the statements on the questionnaire based on their actual evaluation of how often they have been supervised and attitude of staff nurses towards supervision. If the participants had most of the responses strongly agree and agree it meant that the participant was frequently supervised and had help when needed. The four Likert point responses had been opted for and the uncertain category was omitted to ensure that participants made a clear choice on the positives or negatives of clinical learning.

### Validation of the instrument

Validity and reliability of the instrument was evaluated by conducting pilot study. Furthermore, a large sample of 590 was also obtained which covered the targeted groups. In addition, to ensure face validity, experts in nursing education and an expert in statistics reviewed the questionnaire. Their comments were incorporated in the final questionnaire. Some comments required rewording of statements and others were deleted. Moreover, a coefficient reliability test using Cronbach’s alpha was carried out to determine internal consistency of the items in the instrument if they were reliable in measuring the dependent variables. Cronbach alpha coefficient was conducted for each of the sub scales of clinical supervision which was 0.8. However, some of the sub scales have been previously tested and used in other countries including Malaysia, [3] this was for local validation.

### Data collection

Data was collected over a period of ten months from October 2013 to July 2014 using self-administered questionnaire. Two research assistants who were university graduates with some experiences in data collection assisted in data collection. The students were asked to assemble in a class where questionnaires were handed out for them to fill in and were collected after they had been completed. This arrangement helped to increase the response rate. Each questionnaire took 20–30 minutes for the participants to complete. Quantitative data was collected from 590 participants: College 1 (n = 89), College 2 (n = 36), College 3 (n = 49), college 4 (n = 68), College 5 (n = 69), College 6 (n = 104), College 7 (n = 64) and College 8 (n = 111).

### Data analysis

Quantitative data was analysed using Statistical Package for Social Sciences (SPSS) computer software (version 22.0). Univariate analysis was then used to measure socio-demographic characteristics of participants. Bivariate analysis was performed using one-way Analysis of Variance (ANOVA) to examine the association between clinical supervision variable and independent variables; namely programmes, colleges and years of study, hospital, wards/units and duration of placement and number of times students met with nurse educators. A p-value was used to test statistical significance and the level of confidence was p ≤ 0.05.

Atlas ti (version 7) was used to analyse data collected from focus groups discussions. This study comprised of sixteen focus groups, two focus groups from each college, with nine students per group three from each year of study which were selected through purposive sampling and a total of 144 students participated in this study through focus group discussions. The groups consisted of male and female students of different age groups and year of study with different experiences. The discussions were tape recorded after obtaining permission from the participants.

In this study all principles of rigor were observed. Some participants were shown the summary of the findings to confirm whether it was a true reflection of their perceptions and experiences. The researcher also presented the findings to peers for review of any biases and misinterpretations on the analysis thus increasing the credibility of the study. In addition, a prolonged engagement with the participants and the data was done in order to ensure understanding of their views and opinions through questioning and clarification.

### Ethical consideration

Ethical clearance was granted from North West University Committee for Research on Human Subjects in South Africa and the National Health Science Research Committee, Ministry of Health in Malawi. Permission to conduct the study was also obtained from the colleges. Oral and written consent to conduct the study was obtained from participants after explaining and giving them information sheet on the purpose of the study, the process of data collection and their role and that participation was entirely voluntary. Confidentiality and anonymity were also assured as well as right to withdraw at any time in the research process. The participants voluntarily consented to participate in the study. In addition, permission to obtain information on caseloads and staffing in the clinical setting was also sought and was given.

### Study limitations

The experiences of clinical staff and nurse educators could also have been obtained, which would have provided different views and opinions regarding supervision of students in the clinical setting. Additionally, only students placed at central hospital were included. Despite these limitations the results are of considerable importance for nursing education especially in clinical teaching and learning in Malawi.

## Questionnaire Findings

### Clinical supervision students received

Table 1 shows that RN diploma programme had the highest number 84.7% (n = 72) of participants who received clinical supervision. The results indicated that there was no statistical significant difference on clinical supervision of students and the programme of study (F 2, 587) 2.479, p > 0.085).

**Table 1:** Clinical supervision by programmes, institutions and levels of study.

Programmes	Received Clinical supervision	
	Yes	No

	N (%)	N (%)

Bsc Nursing	66 (74.2)	23 (25.8)
RN Diploma	72 (84.7)	13 (15.3)
Enrolled (NMT)	305 (73.3)	111 (26.7)
**Institutions**		

College 1	66 (74.2)	23 (25.8)
College 2	31 (86.1)	5 (13.9)
College 3	41 (83.7)	8 (16.3)
College 4	50 (73.5)	18 (26.5)
College 5	45 (65.2)	24 (34.8)
College 6	60 (57.7)	44 (42.3)
College 7	59 (92.2)	5 (7.8)
College 8	91 (82.0)	20 (18.0)
**Levels of study**		

First year	124 (80.5)	30 (19.5)
second year	160 (69.6)	70 (30.4)
Third year	159 (77.2)	47 (22.8)

N = Number, % = percentage,* = p < 0.05, ** = p < 0.001.

### Clinical supervision by institution of study

Table 1 displays that the majority 92.2% (n = 59) of participants from college 7 received clinical supervision; with college 6 having the highest proportion 42.3% (n = 44) of participants who did not receive clinical supervision. Table 2 displays the significant association between institution of study and clinical supervision (F 7,582) 5.665, p < 0.00A1). Scheffe’s post hoc test revealed significant differences occurred between college 6 and college 7 (\bar x) 0.3450, p < 0.001 and between college 6 and college 8 (\bar x) –0.2430, p < 0.014 (Table 4.19). Participants from college 6 received less supervision compared to those from college 1.

**Table 2:** Scheffe’s post hoc multiple comparisons on institution of study and clinical supervision.

Institutions		\boldsymbol{(\overline{x})}		p	CI	F-statistics

College 6	College 1	–0.1647		0.398	[–0.3937–0.0644]	5.665**
	College 2	–0.2842		0.098	[–0.5910–0.0226]	
	College 3	–0.2598		0.083	[–0.5347–0.0151]	
	College 4	–0.1584		0.563	[–0.4058–0.0891]	
	College 5	–0.0753		0.988	[–0.3216–0.1711]	
	College 7	0.3450	**	0.001	[–0.5970–0.0929]	
	College 8	–0.2429	*	0.014	[–0.4594–0.0264]	

College 7	College 1	0.1803		0.449	[–0.0797–0.4403]	
	College 2	0.0608		––	[–0.2698–0.3913]	
	College 3	0.0852		0.992	[–0.2160–0.3863]	
	College 4	0.1866		0.488	[–0.0897–0.4629]	
	College 5	0.2697		0.061	[–0.0056–0.5450]	
	College 6	0.3450	**	0.001	[0.0929–0.5970]	
	College 8	0.1021		0.935	[–0.1470–0.3511]	

College 8	College 1	0.0783		0.974	[–0.1475–0.3040]	
	College 2	–0.0413		––	[–0.3456–0.2630]	
	College 3	–0.0169		––	[–0.2890–0.2552]	
	College 4	0.0845		0.974	[–0.1598–0.3288]	
	College 5	0.1677		0.458	[–0.0756–0.4109]	
	College 6	0.2429	*	0.014	[0.0264–0.4594]	
	College 7	–0.1021		0.935	[–0.3511–0.1470]	

* = significant at p < 0.05, ** p < 0.001, (\bar x) = Mean Difference, p = Significance CI = 95% Confidence Interval.

### Clinical supervision by hospital of placement

Table 3 shows that the majority (86%, n = 31) of students who received supervision were those placed at hospital B. Results showed that hospital of placement was statically significant with clinical supervision received (F3, 586) = 3.714, p < 0.011). The significant differences existed between hospital C and hospital D (\bar x) –0.1391, p < 0.038.

**Table 3:** Scheffe’s post hoc multiple comparisons on hospital of placement and clinical supervision.

Hospital		\boldsymbol{(\overline{x})}		p	CI	F-statistics

Hospital A	Hospital B	–0.1278		0.464	[–0.3515–0.0959]	3.714*
	Hospital C	0.0244		0.956	[–0.0967–0.1455]	
	Hospital D	–0.1149		0.207	[–0.2654–0.0356]	

Hospital B	Hospital A	0.1278		0.464	[–0.0959–0.3515]	
	Hospital C	0.1522		0.261	[–0.0607–0.3651]	
	Hospital D	0.0129		0.999	[–0.2180–0.2438]	

Hospital C	Hospital A	–0.0244		0.956	[–0.1455–0.0967]	
	Hospital B	–0.1522		0.261	[–0.3651–0.0607]	
	Hospital D	–0.1393	**	0.038	[–0.2733–0.0053]	

Hospital D	Hospital A	0.1149		0.207	[–0.0356–0.2654]	
	Hospital B	–0.0129		0.999	[–0.2438–0.2180]	
	Hospital C	0.1393	**	0.038	[0.0053–0.2733]	

* = Significant at p < 0.05, ** p < 0.001, (\bar x) = Mean Difference, p = Significance CI = 95% Confidence Interval.

#### Clinical supervision by number of times students met Nurse Educator (NE)

Table 4 shows that about 85% (n = 58) of participants who met with the NE seven (7) times and above received supervision. There was significant relationship between clinical supervision and number of times students met with the NE F (4, 585), 6.399, p < 0.001). Scheffe’s statistics indicated significant differences between students who did not meet the NE and those who met the NE 1–2 times (\bar x) 0.179, p < 0.026, 3–4 times (\bar x) 0.254, p < 0.001, 5–6 times (\bar x) 0.223, p < 0.036 and 7 times and above (\bar x) 0.291, p < 0.001. Thus, participants who received clinical supervision were those who met the NE regardless of frequency.

**Table 4:** Scheff’s post hoc multiple comparisons on number of times students met with Nurse Educator (NE) and clinical supervision.

Times met with NE		\boldsymbol{(\overline{x})}		p	CI

Not met with NE	Met 1–2 times	–0.1789	*	0.026	[–0.3444 – –0.0135]
	Met 3–4 times	–0.2540	**	0.001	[–0.4293 – –0.0786]
	Met 5–6 times	–0.2229	*	0.036	[–0.4372 – –0.0085]
	Met 7 times above	–0.2911	**	0.001	[–0.5028 – –0.0795]
	Not met	0.1790	*	0.026	[0.0135–0.3444]

Met NE 1–2 times	Met 3–4 times	–0.0751		0.596	[–0.2141–0.0640]
	Met 5–6 times	–0.0439		0.970	[–0.2297–0.1420]
	Met 7 times above	–0.1122		0.463	[–0.2949–0.0705]

Met NE 3–4 times	Not met	0.0568		0.001	[0.0786–0.4293]
	Met 1–2 times	0.0450		0.596	[–0.0640–0.2141]
	Met 5–6 times	0.0630		0.993	[–0.1635–0.2259]
	Met 7 times above	0.0620		0.986	[–0.2288–0.1545]

Met NE 5–6 times	Not met	0.2228	*	0.036	[0.0085–0.4372]
	Met 1–2 times	0.0439		0.970	[–0.1420–0.2297]
	Met 3–4 times	–0.0312		0.993	[–0.2259–0.1635]
	Met 7 times above	–0.0683		0.930	[–0.2962–0.1596]

Met NE 7 times above	Not met	0.2911	**	0.001	[0.0795–0.5028]
	Met 1–2 times	0.1122		0.463	[–0.0705–0.2949]
	Met 3–4 times	0.0372		0.986	[–0.1545–0.2288]
	Met 5–6 times	0.0683		0.930	[–0.1596–0.2962]

**p < 0.05, ***p < 0.001, F = 6.399***, (\bar x) = Mean Difference, p = Significance CI = 95% Confidence Interval.

### Satisfaction with clinical supervision

Satisfaction with clinical supervision and programme of study was found to be significant F (2,587) 3.541, p < 0.030). Table 5 shows that participants from BSc programme were less satisfied with clinical supervision than those in RN Diploma (\bar x) –0.1454, p < 0.047 whereas satisfaction with clinical supervision was the same between BSc and NMT programmes (p 0.069).

**Table 5:** Scheffe’s post hoc multiple comparisons on programme of study and satisfaction with clinical supervision.

Programme of study		\boldsymbol{(\overline{x})}		p	CI

BSc Nursing	RN Diploma	–0.1454	*	0.047	[–0.2893 – –0.0015]
	Enrolled (NMT)	–0.1042		0.069	[–0.2157–0.0060]

RN Diploma	BSc Nursing	0.1454	*	0.047	[0.0015–0.2893]
	Enrolled (NMT)	0.0406		0.678	[–0.0724–0.1536]

Enrolled (NMT)	BSc Nursing	0.1048		0.069	[–0.0060–0.2157]
	RN Diploma	–0.0406		0.678	[–0.1536–0.0724]

* = significant at p < 0.05, **p < 0.001, (\bar x) = Mean Difference, p = Significance CI = 95% Confidence Interval.

Although there was a significant relationship between institutions and satisfaction (F 7, 582) 2.642, p < 0.011), there was no significant relationship between the institutions of study and clinical supervision. Clinical supervision between the institutions was the same.

### Focus group findings

Contrary to quantitative findings, the qualitative aspect revealed that supervision by NE and clinical staff (preceptors) was also found to be inadequate. The majority of students indicated having difficulties in finding help when needed, especially when confronted with difficult situations. It was indicated that the NE infrequently went to the clinical area, mainly only for clinical assessments and orientation of students going to wards for first time.

The following themes arose from the discussion: lack of clinical teaching, guidance and support, lack of human resources and learning support, job insecurity, and lack of remuneration as reasons for lack of supervision, role models, and student guidance despite pressure and self-directed.

### Lack of clinical teaching, guidance and support

Lack of clinical teaching and support in the clinical area came out frequently in the discussions. It was evident that the NEs would only come for orientation of students, for clinical assessments and sometimes for evaluation of their case studies. However, students were not guided or observed by the clinical staff or the NE when performing procedures so that they would know if learning is taking place as illustrated in the excerpts:

“Sometimes an allocation maybe for three months you may be visited only once and the time when they are doing assessments otherwise you will not see any tutor or follow up on whatever you are doing I think we are lacking when it comes to learning”. (C5 Y2)

### Lack of human resources and learning support

It appeared that the students were mainly with clinical staff who were not willing to teach and help them. It was clear that students would have liked their NEs to be coming to the clinical area to teach them as the following excerpt illustrates:

“I was supposed to do tracheotomy care. There wasn’t any clinical instructor apart from the nurses on duty. I asked one of the nurses on duty to supervise me. He told me “Go and do it I will sign because I have other things to do””. (C2 Y3)

It was apparent from the participants that the students were not guided or observed by the clinical staff and the NEs when performing procedures for learning to take place. It was evident from the participants that shortage of NEs also contributed to the problem of lack of supervision. Students had difficulties to find help when needed.

“We lack of guidance and teaching because when we were sent in the clinical area our tutors do not visit us frequently and as a result, compromising our learning”. (C3 Y2)

The discussions revealed that first year participants who were in the clinical setting for the first time lacked support and being new in the profession they needed clinical staff and NEs to guide and supervise them to do basic tasks. They had to wait for preceptors and NEs to be able to perform skills:

“As the first year student I expected to learn much of our work is bed making, taking vital signs but there was nobody to teach us”. (C1 Y1)

From all the focus group sessions it was evident that students were learning without support and guidance. It was observed from the discussions that students were sometimes left alone in wards or units, even in placements which require them to be under strict supervision, including the labour ward and Intensive Care Unit (ICU), as a result they would be stranded if a patient’s condition changes. These sentiments were expressed thus:

“………We are just left alone especially when we are in labour ward which is not good”……. (C6 Y3)

### Job insecurity and lack of remuneration as reasons for lack of supervision

It was noted from the discussions that clinical staff did not want to teach students because they were not being paid for that, were overloaded with work and they were not employed to teach students which was perceived as the duty of the NEs. In addition, it was noticeable that qualified NMTs were reluctant to supervise the BSc students as they would become seniors to them once they qualify. The qualified NMTs were also unwilling to teach NMT students as it was perceived that they would be at the same level. This was indicated to be affecting students’ clinical learning and eventually the profession as a whole as pointed out by participants:

“Some clinical staff just looked at us without helping us and when you ask them they just said “You know your tutors get paid for supervising and you want me to supervise you without getting paid? I cannot do that” so it was really affecting us”. (C4 Y3)

### Role models and student guidance despite pressure

It is worth noting from the discussions that some students indicated that despite students lacking support and supervision from the majority of NE and clinical staff, there were a few who were willing to teach them. These were not only eager to teach but were always available for the students, despite their increasing workload:

“I was not conversant with cannulation I asked the staff nurse who was kind and approachable to assist me she helped and made sure I knew the skill”. (C8 Y2)

However, it was noted from the discussions that some of the NEs were also willing to teach. These were role models and were found to even teach students from other colleges by demonstrating skills to them. Students felt that if there were several of that kind of the NEs, they would see some changes in learning in clinical areas.

“When you talk about our tutors, they do their best. At least when they come they target 2 to 3 students, teach those ones so that they become competent although sometimes they do not come due to other programs. There are somewhere with other students and some are in class but otherwise they really help us”. (C4 Y1)

## Discussion

The aim of this study was to explore and describe experiences of pre-registration nursing students on clinical supervision. The importance of clinical supervision in clinical practice for nursing students to develop into nursing professionals cannot be underestimated [10]. Students require support and guidance from those who are professional experts in the clinical setting for personal and professional growth. However, this role is not fulfilled, as students lack supervision [11,20].

Quantitative results showed that students received clinical supervision. Contrary to the quantitative findings, from the qualitative perspective, it implies that students were not receiving adequate clinical supervision from both NE and clinical staff (preceptors). The majority of students indicated having difficulties in finding help when it was needed, especially when confronted with difficult situations. Therefore, from both quantitative and qualitative results it can be concluded that although students received clinical supervision it was inadequate and irregular for learning to take place.

Similar findings were reported in other studies where students lacked support and supervision from nurse teachers and preceptors [11,18]. The nurse educators rarely went to the clinical area to supervise students as they would finish an allocation without being supervised. Similarly, in a study of European countries [25] namely: Belgium, Cyprus, Finland, Ireland, Italy, the Netherlands, Spain, Sweden and the United Kingdom (UK), found that 13% of students did not meet with nurse teachers. This was different from another study [18], as supervision by teachers was scored higher. The differences in the findings could be due to different settings. In the current study NEs went to the clinical setting infrequently, mainly for orientation in the new allocation and for clinical assessments. Similar findings were reported in Iran [26], students indicated that clinical instructors were in the clinical setting mainly for evaluation as opposed to supervision. The importance of nurse educators in the clinical supervision of students cannot be underestimated as they know students’ expected outcomes. Therefore, they need to provide guidance and support to both the students and the preceptors [8]. The unavailability of NEs in the clinical setting in the current study can be explained by the shortage of NEs and increased responsibilities on them. NEs have to supervise students in the clinical area but also teach other students theory in the classroom, apart from holding other responsibilities. In this study, being supervised once during orientation and finishing an allocation without supervision is an indication of inadequate and lack of supervision.

This study revealed that students from college 6 received less supervision compared to other institutions. This may be related to the hospital of placement. The results showed that clinical supervision was significantly associated with hospital of placement and students placed at hospital C were less likely to receive clinical supervision compared to those placed at hospital D (bigger hospital with more services compared to hospital C). Thus, with College 6 being close to hospital C, the majority of students may be allocated to this hospital. If students lack supervision and support they may lose confidence, be unable to properly learn the practice of nursing, be unable to develop decision making and problem solving skills, and thereafter become unsafe to patients [2,11,17].

Although in this study show that students received clinical supervision at one point during placement they were dissatisfied with their clinical supervision. Satisfaction with clinical learning depends on clinical supervision received in the clinical area. Students are satisfied with clinical supervision if they are supervised more often and receive individualized supervision not group supervision [28]. Contrary to this finding, another study [15], students were satisfied with clinical supervision. The reason could be that students were frequently supervised by both preceptors and teachers enabling them to achieve their learning outcomes. The differences could be due to infrequency supervision in this study. The students in the BSc programme were more dissatisfied with clinical supervision compared with those from other programmes. Students in the BSc programme may have met the nurse educators less frequently as satisfaction with clinical supervision was found to be related to frequency of supervision. In addition, it could have been related to reluctance of the clinical staff, the majority of whom were NMTs, to supervise the BSc students, saying they could not supervise students who would qualify at a higher level than them upon completing their education. Consistent with these findings were the findings of two studies [20,7] as the status inferiority of qualified staff of lower educational level affected students’ clinical supervision negatively. These findings were however contrary to those by another study [13] in Malawi, where students were satisfied with clinical supervision. The inconsistency may be due to this [13] study being conducted at a hospital-based college while for this current study, students were placed in referral hospitals where supervision may be inadequate due to increased workload. A study in Norway showed student satisfaction with clinical supervision by nurse teachers and preceptors [18]. The difference in findings between that study and the current one could be due to type of clinical supervision as in Norway they used one to one mentorship supervision. Additionally, Norway being a developed country, there may be adequate staff and fewer caseloads.

Additionally, students in this study were not supervised individually as a result their needs were not met. Individualized supervision facilitates learning as the one to one relationship with the mentor or preceptor allows students to develop self-confidence, professionally, independence and promote role socialization thereby attaining clinical competency [22,28].

Findings from this study reveal that the students perceived that inadequate supervision was as a result of too many students in the clinical area, and shortage of NEs and clinical staff to adequately supervise students. In addition, heavy workloads and busy wards/units, led to staff not having ample time to perform both roles of patient care and student teaching. Such a situation where staff performs dual roles has been found to compromise the fulfillment of their roles as preceptors [11] (Hallin and Danielson, 2009). Clinical performance increases if students are given the necessary support in the clinical environment [6].

Clinical supervision reduces the theory-practice gap, enhances team work and promotes personal and professional development and patient safety. In addition, it enhances students’ acquisition of professional skills in becoming competent practitioners [1,23]. Lack of adequate clinical supervision may cause students to become demotivated, unable to attain learning outcomes, lose confidence and even leave the profession [18].

## Conclusion

The study findings show that students were not adequately supervised by both clinical nurses and Nurse Educators. This was attributed to negative attitudes of staff towards clinical supervision of students, shortage of staff and nurse educators, congestion of students in the clinical area and heavy workloads. It was also found that the college where students were coming from and the hospital of placement had a relationship on the quality of clinical supervision received. Generally, the students were not satisfied with clinical supervision as they were infrequently supervised, lacked help when needed and the supervision was not individualized. This may indicate that students were unable to effectively learn the skills of the nursing profession thus being incompetent and unsafe to patients as there was no role modeling, direction and guidance.

## Policy implications and options

Findings from this study may have implications on the practice of nursing education in Malawi. There is a need to revisit the number of students enrolled in nursing colleges to ensure that the quality of clinical learning is not compromised.

The following recommendations are therefore suggested:

- Nursing education institutions need to provide students with supervisory support. One option would be for the institutions to employ retired nurses on a part time basis to supervise students.
- Nursing education institutions need to provide training and support to clinical staff on how to supervise students in the clinical setting so that they know what is expected of them in regard to student supervision.
- There is a need for building the capacity of clinical supervisors and addressing the issues of supervision is essential.
- Nurse educators should provide continuous support and guidance to the students. They should work in the clinical area with students as part of the health care team.
- There is a need for clinical staff to be aware of their role in clinical education and the need to fit clinical teaching in their schedules of patient care so that students are adequately supervised.

## Further Research

The following areas of research for the future are recommended.

Thus, a further research would be recommended on clinical supervision as viewed by clinical nurses, Nurse Educators and those students placed in district hospitals.
